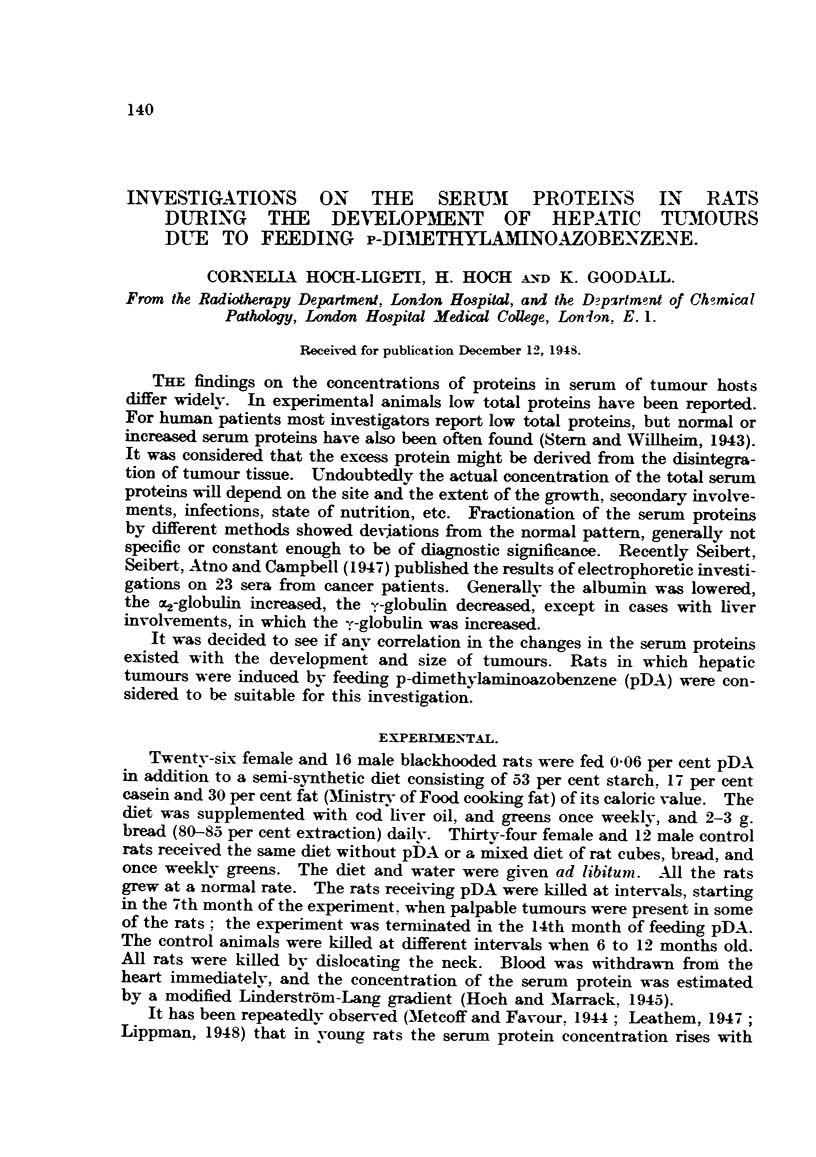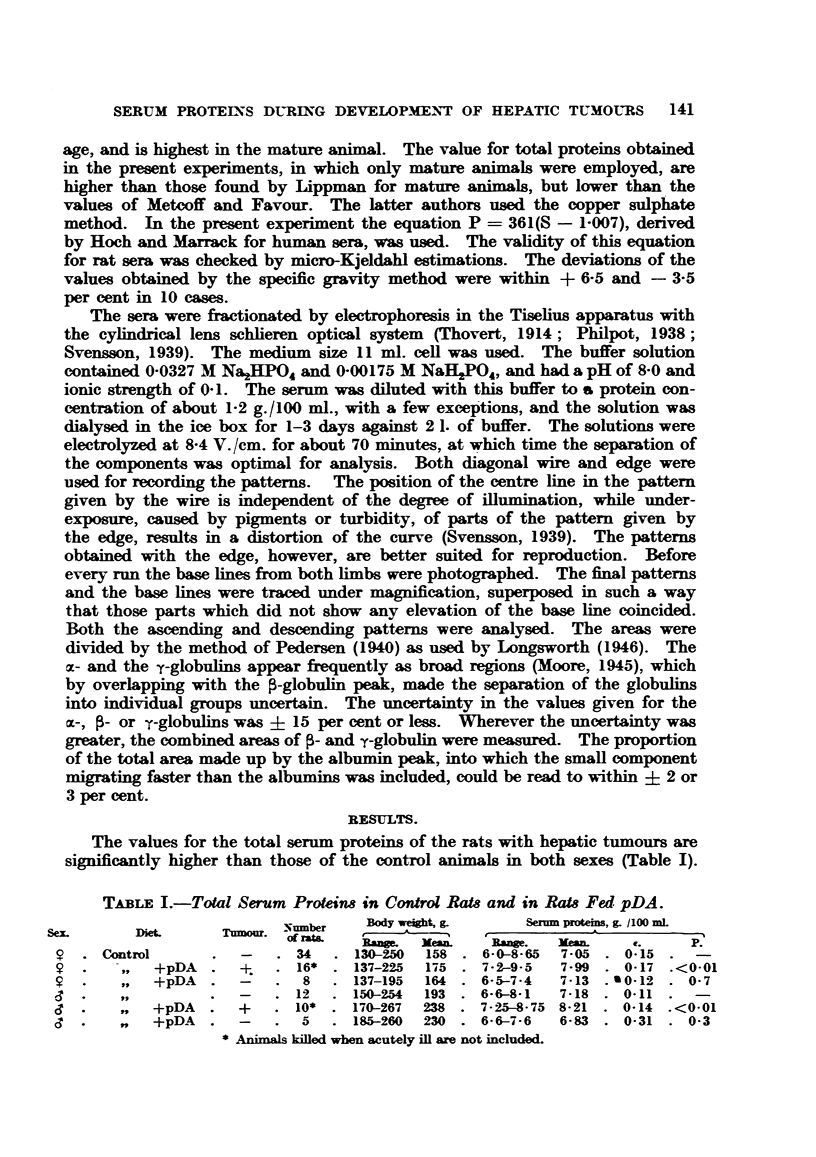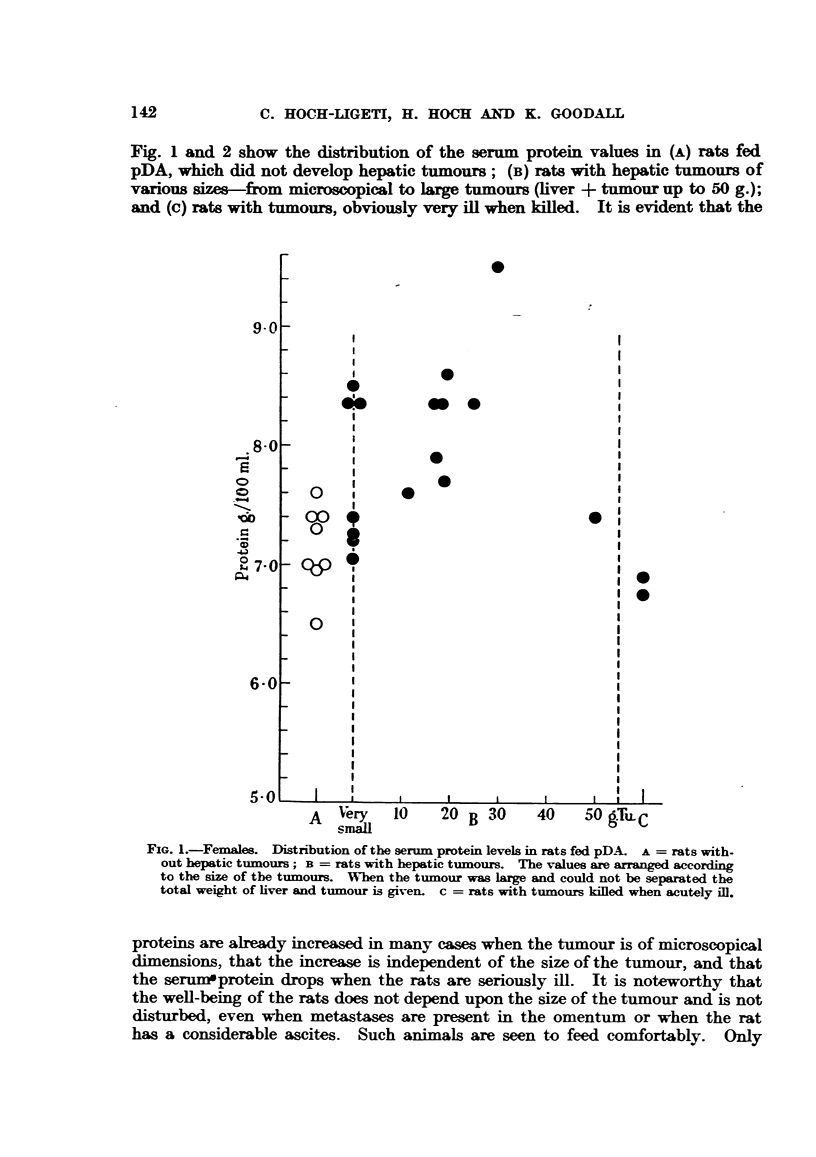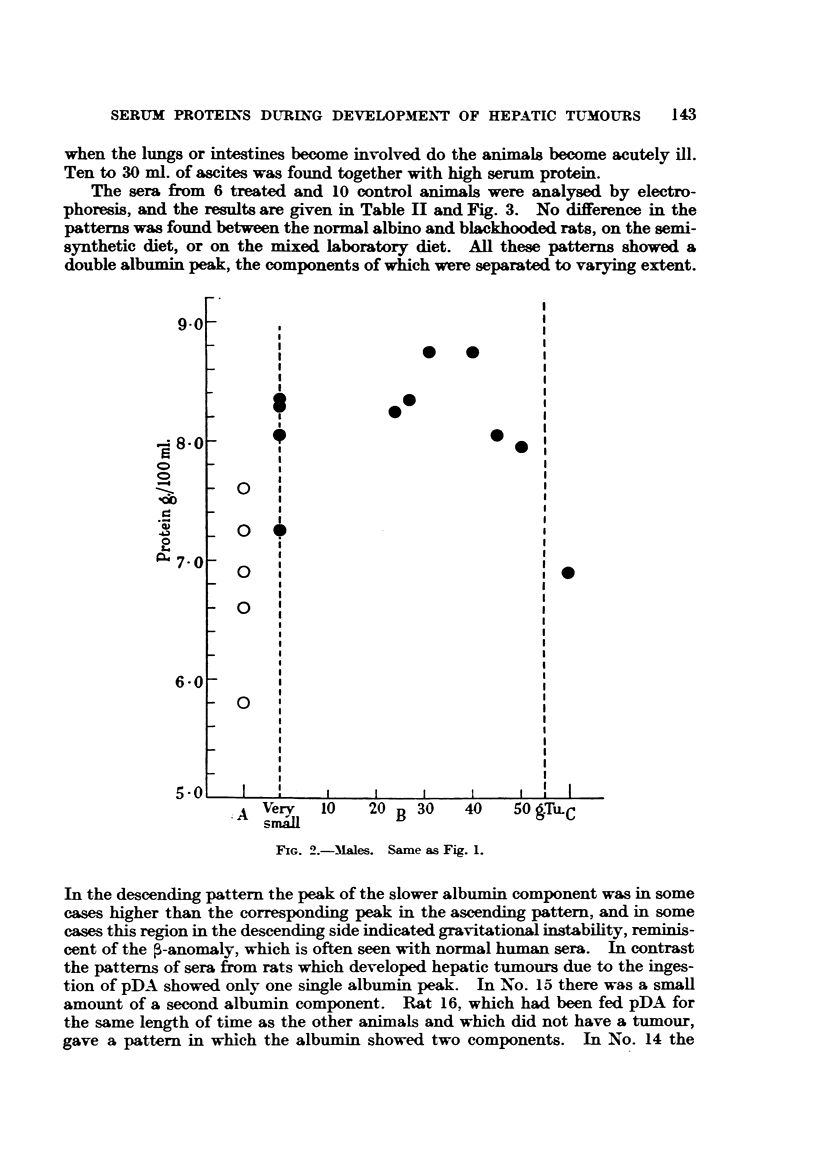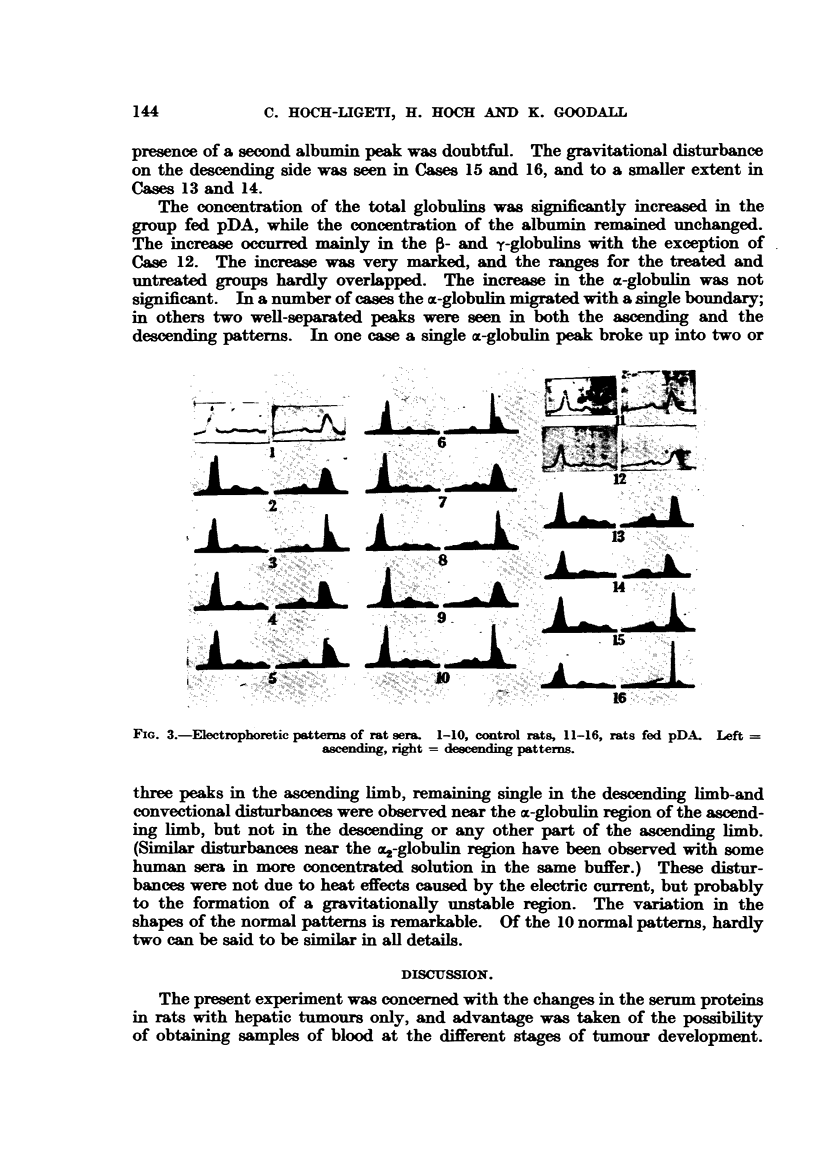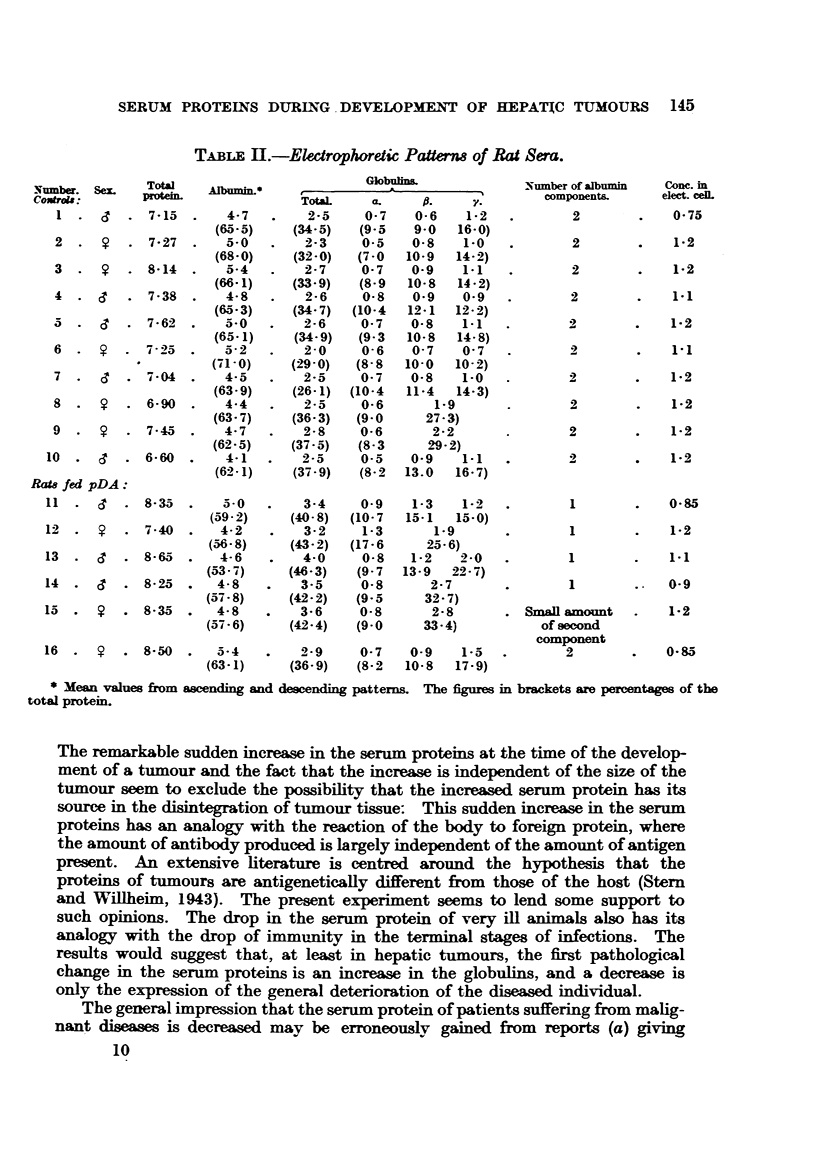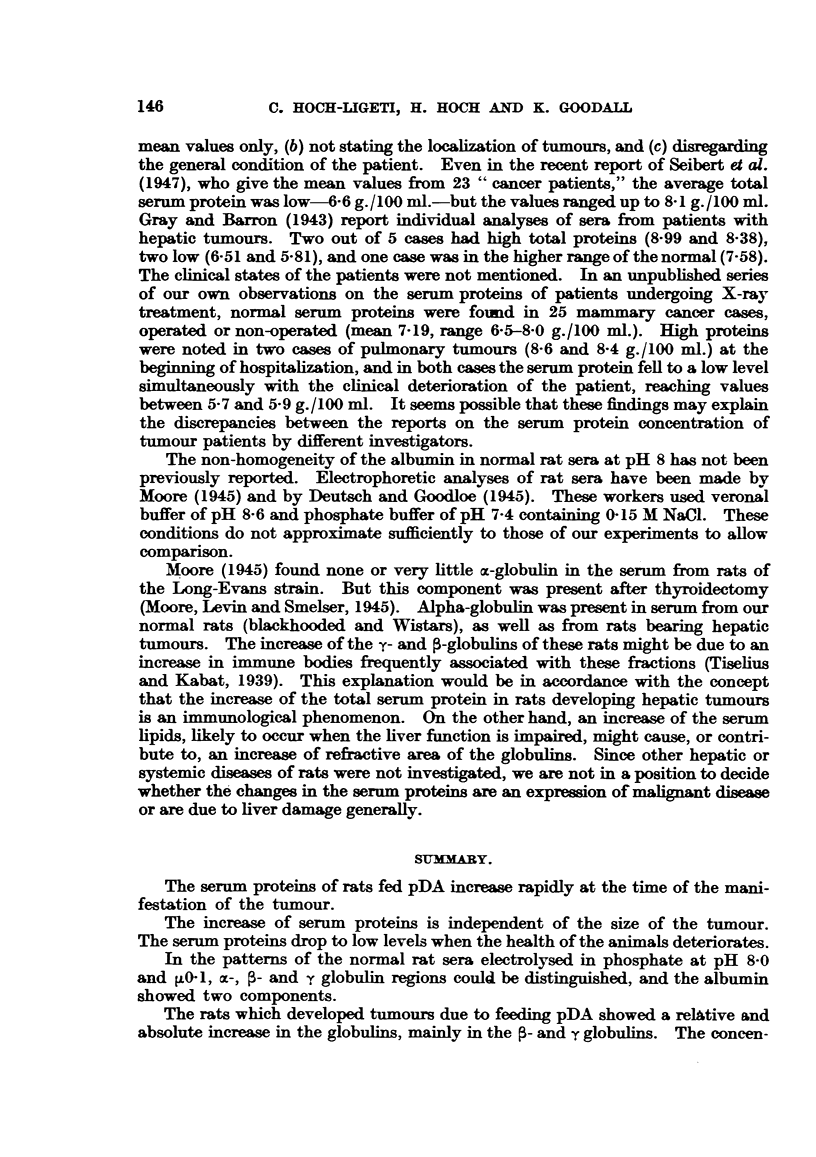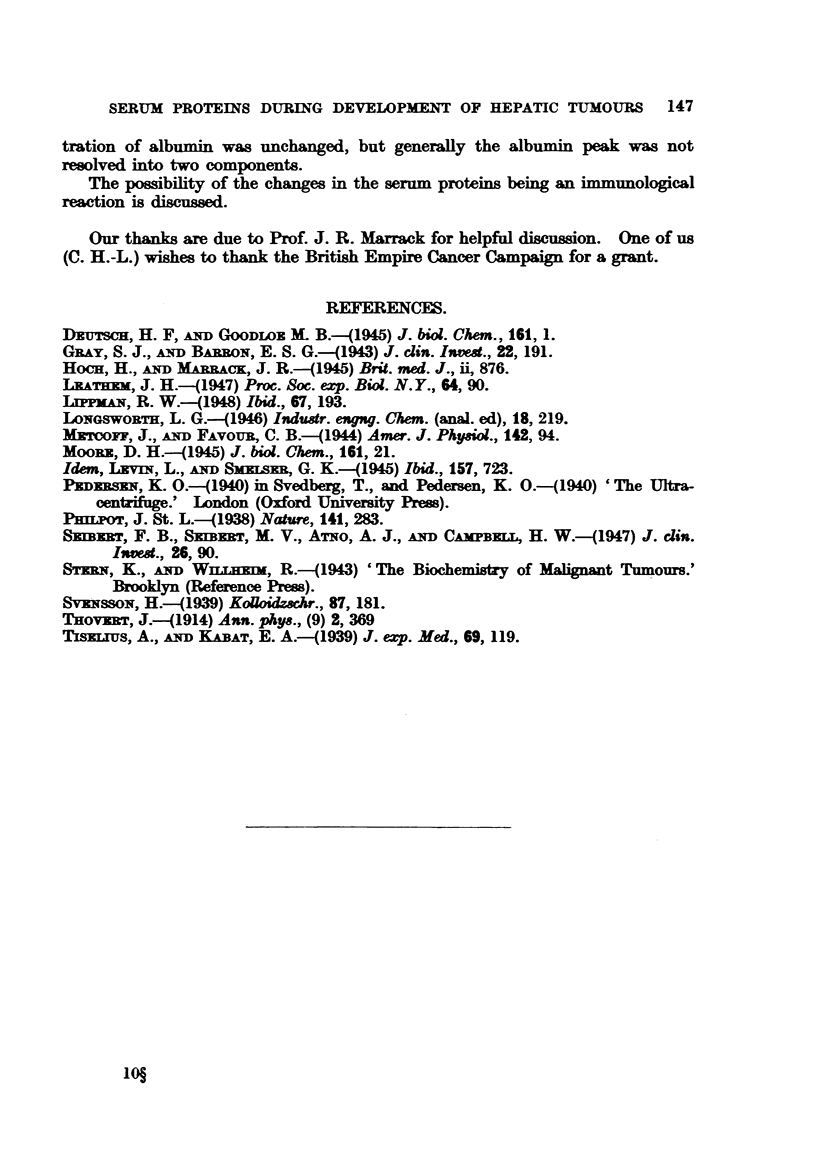# Investigations on the Serum Proteins in Rats during the Development of Hepatic Tumours due to Feeding p-Dimethylaminoazobenzene

**DOI:** 10.1038/bjc.1949.16

**Published:** 1949-03

**Authors:** Cornelia Hoch-Ligeti, H. Hoch, K. Goodall

## Abstract

**Images:**


### INVESTIGATIONS ON THE SERUM PROTEINS IN RATS DURING THE DEVELOPMENT OF HEPATIC TUMOURS DUE TO FEEDING p-DIMETHYLAMINOAZOBENZENE.

CORNELIA HOCH-LIGETI, H. HOCH AND K. GOODALL.

*From the Radiotherapy Department, London Hospital, and the Department of Chemical Pathology, London Hospital Medical College, London, E. 1.*

Received for publication December 12, 1948.

THE findings on the concentrations of proteins in serum of tumour hosts differ widely. In experimental animals low total proteins have been reported. For human patients most investigators report low total proteins, but normal or increased serum proteins have also been often found (Stern and Willheim, 1943). It was considered that the excess protein might be derived from the disintegration of tumour tissue. Undoubtedly the actual concentration of the total serum proteins will depend on the site and the extent of the growth, secondary involvements, infections, state of nutrition, etc. Fractionation of the serum proteins by different methods showed deviations from the normal pattern, generally not specific or constant enough to be of diagnostic significance. Recently Seibert, Seibert, Atno and Campbell (1947) published the results of electrophoretic investigations on 23 sera from cancer patients. Generally the albumin was lowered, the $\alpha_2$-globulin increased, the $\gamma$-globulin decreased, except in cases with liver involvements, in which the $\gamma$-globulin was increased.

It was decided to see if any correlation in the changes in the serum proteins existed with the development and size of tumours. Rats in which hepatic tumours were induced by feeding p-dimethylaminoazobenzene (pDA) were considered to be suitable for this investigation.

### EXPERIMENTAL.

Twenty-six female and 16 male blackhooded rats were fed 0·06 per cent pDA in addition to a semi-synthetic diet consisting of 53 per cent starch, 17 per cent casein and 30 per cent fat (Ministry of Food cooking fat) of its caloric value. The diet was supplemented with cod liver oil, and greens once weekly, and 2–3 g. bread (80–85 per cent extraction) daily. Thirty-four female and 12 male control rats received the same diet without pDA or a mixed diet of rat cubes, bread, and once weekly greens. The diet and water were given *ad libitum*. All the rats grew at a normal rate. The rats receiving pDA were killed at intervals, starting in the 7th month of the experiment, when palpable tumours were present in some of the rats ; the experiment was terminated in the 14th month of feeding pDA. The control animals were killed at different intervals when 6 to 12 months old. All rats were killed by dislocating the neck. Blood was withdrawn from the heart immediately, and the concentration of the serum protein was estimated by a modified Linderström-Lang gradient (Hoch and Marrack, 1945).

It has been repeatedly observed (Metcoff and Favour, 1944 ; Leathem, 1947 ; Lippman, 1948) that in young rats the serum protein concentration rises with age, and is highest in the mature animal. The value for total proteins obtained in the present experiments, in which only mature animals were employed, are higher than those found by Lippman for mature animals, but lower than the values of Metcoff and Favour. The latter authors used the copper sulphate method. In the present experiment the equation $P = 361(S - 1{\cdot}007)$, derived by Hoch and Marrack for human sera, was used. The validity of this equation for rat sera was checked by micro-Kjeldahl estimations. The deviations of the values obtained by the specific gravity method were within $+ 6{\cdot}5$ and $- 3{\cdot}5$ per cent in 10 cases.

The sera were fractionated by electrophoresis in the Tiselius apparatus with the cylindrical lens schlieren optical system (Thovert, 1914; Philpot, 1938; Svensson, 1939). The medium size 11 ml. cell was used. The buffer solution contained 0·0327 M $Na_2HPO_4$ and 0·00175 M $NaH_2PO_4$, and had a pH of 8·0 and ionic strength of 0·1. The serum was diluted with this buffer to a protein concentration of about 1·2 g./100 ml., with a few exceptions, and the solution was dialysed in the ice box for 1–3 days against 2 l. of buffer. The solutions were electrolyzed at 8·4 V./cm. for about 70 minutes, at which time the separation of the components was optimal for analysis. Both diagonal wire and edge were used for recording the patterns. The position of the centre line in the pattern given by the wire is independent of the degree of illumination, while under-exposure, caused by pigments or turbidity, of parts of the pattern given by the edge, results in a distortion of the curve (Svensson, 1939). The patterns obtained with the edge, however, are better suited for reproduction. Before every run the base lines from both limbs were photographed. The final patterns and the base lines were traced under magnification, superposed in such a way that those parts which did not show any elevation of the base line coincided. Both the ascending and descending patterns were analysed. The areas were divided by the method of Pedersen (1940) as used by Longsworth (1946). The α- and the γ-globulins appear frequently as broad regions (Moore, 1945), which by overlapping with the β-globulin peak, made the separation of the globulins into individual groups uncertain. The uncertainty in the values given for the α-, β- or γ-globulins was ± 15 per cent or less. Wherever the uncertainty was greater, the combined areas of β- and γ-globulin were measured. The proportion of the total area made up by the albumin peak, into which the small component migrating faster than the albumins was included, could be read to within ± 2 or 3 per cent.

### RESULTS.

The values for the total serum proteins of the rats with hepatic tumours are significantly higher than those of the control animals in both sexes (Table I).

TABLE I.—*Total Serum Proteins in Control Rats and in Rats Fed pDA.*

| Sex. | Diet. | Tumour. | Number of rats. | Body weight, g. Range. | Body weight, g. Mean. | Serum proteins, g. /100 ml. Range. | Serum proteins, g. /100 ml. Mean. | ε. | P. |
|---|---|---|---|---|---|---|---|---|---|
| ♀ | Control | — | 34 | 130–250 | 158 | 6·0–8·65 | 7·05 | 0·15 | — |
| ♀ | ,, +pDA | + | 16* | 137–225 | 175 | 7·2–9·5 | 7·99 | 0·17 | <0·01 |
| ♀ | ,, +pDA | — | 8 | 137–195 | 164 | 6·5–7·4 | 7·13 | 0·12 | 0·7 |
| ♂ | ,, | — | 12 | 150–254 | 193 | 6·6–8·1 | 7·18 | 0·11 | — |
| ♂ | ,, +pDA | + | 10* | 170–267 | 238 | 7·25–8·75 | 8·21 | 0·14 | <0·01 |
| ♂ | ,, +pDA | — | 5 | 185–260 | 230 | 6·6–7·6 | 6·83 | 0·31 | 0·3 |

* Animals killed when acutely ill are not included.

Fig. 1 and 2 show the distribution of the serum protein values in (A) rats fed pDA, which did not develop hepatic tumours; (B) rats with hepatic tumours of various sizes—from microscopical to large tumours (liver + tumour up to 50 g.); and (C) rats with tumours, obviously very ill when killed. It is evident that the

FIG. 1.—Females. Distribution of the serum protein levels in rats fed pDA. A = rats without hepatic tumours; B = rats with hepatic tumours. The values are arranged according to the size of the tumours. When the tumour was large and could not be separated the total weight of liver and tumour is given. C = rats with tumours killed when acutely ill.

proteins are already increased in many cases when the tumour is of microscopical dimensions, that the increase is independent of the size of the tumour, and that the serum protein drops when the rats are seriously ill. It is noteworthy that the well-being of the rats does not depend upon the size of the tumour and is not disturbed, even when metastases are present in the omentum or when the rat has a considerable ascites. Such animals are seen to feed comfortably. Only when the lungs or intestines become involved do the animals become acutely ill. Ten to 30 ml. of ascites was found together with high serum protein.

The sera from 6 treated and 10 control animals were analysed by electrophoresis, and the results are given in Table II and Fig. 3. No difference in the patterns was found between the normal albino and blackhooded rats, on the semi-synthetic diet, or on the mixed laboratory diet. All these patterns showed a double albumin peak, the components of which were separated to varying extent.

Fig. 2.—Males. Same as Fig. 1.

In the descending pattern the peak of the slower albumin component was in some cases higher than the corresponding peak in the ascending pattern, and in some cases this region in the descending side indicated gravitational instability, reminiscent of the β-anomaly, which is often seen with normal human sera. In contrast the patterns of sera from rats which developed hepatic tumours due to the ingestion of pDA showed only one single albumin peak. In No. 15 there was a small amount of a second albumin component. Rat 16, which had been fed pDA for the same length of time as the other animals and which did not have a tumour, gave a pattern in which the albumin showed two components. In No. 14 the presence of a second albumin peak was doubtful. The gravitational disturbance on the descending side was seen in Cases 15 and 16, and to a smaller extent in Cases 13 and 14.

The concentration of the total globulins was significantly increased in the group fed pDA, while the concentration of the albumin remained unchanged. The increase occurred mainly in the β- and γ-globulins with the exception of Case 12. The increase was very marked, and the ranges for the treated and untreated groups hardly overlapped. The increase in the α-globulin was not significant. In a number of cases the α-globulin migrated with a single boundary; in others two well-separated peaks were seen in both the ascending and the descending patterns. In one case a single α-globulin peak broke up into two or

FIG. 3.—Electrophoretic patterns of rat sera. 1–10, control rats, 11–16, rats fed pDA. Left = ascending, right = descending patterns.

three peaks in the ascending limb, remaining single in the descending limb-and convectional disturbances were observed near the α-globulin region of the ascending limb, but not in the descending or any other part of the ascending limb. (Similar disturbances near the $\alpha_2$-globulin region have been observed with some human sera in more concentrated solution in the same buffer.) These disturbances were not due to heat effects caused by the electric current, but probably to the formation of a gravitationally unstable region. The variation in the shapes of the normal patterns is remarkable. Of the 10 normal patterns, hardly two can be said to be similar in all details.

DISCUSSION.

The present experiment was concerned with the changes in the serum proteins in rats with hepatic tumours only, and advantage was taken of the possibility of obtaining samples of blood at the different stages of tumour development.

TABLE II.—*Electrophoretic Patterns of Rat Sera.*

| Number. | Sex. | Total protein. | Albumin.* | Globulins. | | | | Number of albumin components. | Conc. in elect. cell. |
|---|---|---|---|---|---|---|---|---|---|
| | | | | Total. | α. | β. | γ. | | |
| *Controls:* | | | | | | | | | |
| 1 | ♂ | 7·15 | 4·7 | 2·5 | 0·7 | 0·6 | 1·2 | 2 | 0·75 |
| | | | (65·5) | (34·5) | (9·5 | 9·0 | 16·0) | | |
| 2 | ♀ | 7·27 | 5·0 | 2·3 | 0·5 | 0·8 | 1·0 | 2 | 1·2 |
| | | | (68·0) | (32·0) | (7·0 | 10·9 | 14·2) | | |
| 3 | ♀ | 8·14 | 5·4 | 2·7 | 0·7 | 0·9 | 1·1 | 2 | 1·2 |
| | | | (66·1) | (33·9) | (8·9 | 10·8 | 14·2) | | |
| 4 | ♂ | 7·38 | 4·8 | 2·6 | 0·8 | 0·9 | 0·9 | 2 | 1·1 |
| | | | (65·3) | (34·7) | (10·4 | 12·1 | 12·2) | | |
| 5 | ♂ | 7·62 | 5·0 | 2·6 | 0·7 | 0·8 | 1·1 | 2 | 1·2 |
| | | | (65·1) | (34·9) | (9·3 | 10·8 | 14·8) | | |
| 6 | ♀ | 7·25 | 5·2 | 2·0 | 0·6 | 0·7 | 0·7 | 2 | 1·1 |
| | | | (71·0) | (29·0) | (8·8 | 10·0 | 10·2) | | |
| 7 | ♂ | 7·04 | 4·5 | 2·5 | 0·7 | 0·8 | 1·0 | 2 | 1·2 |
| | | | (63·9) | (26·1) | (10·4 | 11·4 | 14·3) | | |
| 8 | ♀ | 6·90 | 4·4 | 2·5 | 0·6 | 1·9 | | 2 | 1·2 |
| | | | (63·7) | (36·3) | (9·0 | 27·3) | | | |
| 9 | ♀ | 7·45 | 4·7 | 2·8 | 0·6 | 2·2 | | 2 | 1·2 |
| | | | (62·5) | (37·5) | (8·3 | 29·2) | | | |
| 10 | ♂ | 6·60 | 4·1 | 2·5 | 0·5 | 0·9 | 1·1 | 2 | 1·2 |
| | | | (62·1) | (37·9) | (8·2 | 13.0 | 16·7) | | |
| *Rats fed pDA:* | | | | | | | | | |
| 11 | ♂ | 8·35 | 5·0 | 3·4 | 0·9 | 1·3 | 1·2 | 1 | 0·85 |
| | | | (59·2) | (40·8) | (10·7 | 15·1 | 15·0) | | |
| 12 | ♀ | 7·40 | 4·2 | 3·2 | 1·3 | 1·9 | | 1 | 1·2 |
| | | | (56·8) | (43·2) | (17·6 | 25·6) | | | |
| 13 | ♂ | 8·65 | 4·6 | 4·0 | 0·8 | 1·2 | 2·0 | 1 | 1·1 |
| | | | (53·7) | (46·3) | (9·7 | 13·9 | 22·7) | | |
| 14 | ♂ | 8·25 | 4·8 | 3·5 | 0·8 | 2·7 | | 1 | 0·9 |
| | | | (57·8) | (42·2) | (9·5 | 32·7) | | | |
| 15 | ♀ | 8·35 | 4·8 | 3·6 | 0·8 | 2·8 | | Small amount of second component | 1·2 |
| | | | (57·6) | (42·4) | (9·0 | 33·4) | | | |
| 16 | ♀ | 8·50 | 5·4 | 2·9 | 0·7 | 0·9 | 1·5 | 2 | 0·85 |
| | | | (63·1) | (36·9) | (8·2 | 10·8 | 17·9) | | |

* Mean values from ascending and descending patterns. The figures in brackets are percentages of the total protein.

The remarkable sudden increase in the serum proteins at the time of the development of a tumour and the fact that the increase is independent of the size of the tumour seem to exclude the possibility that the increased serum protein has its source in the disintegration of tumour tissue. This sudden increase in the serum proteins has an analogy with the reaction of the body to foreign protein, where the amount of antibody produced is largely independent of the amount of antigen present. An extensive literature is centred around the hypothesis that the proteins of tumours are antigenetically different from those of the host (Stern and Willheim, 1943). The present experiment seems to lend some support to such opinions. The drop in the serum protein of very ill animals also has its analogy with the drop of immunity in the terminal stages of infections. The results would suggest that, at least in hepatic tumours, the first pathological change in the serum proteins is an increase in the globulins, and a decrease is only the expression of the general deterioration of the diseased individual.

The general impression that the serum protein of patients suffering from malignant diseases is decreased may be erroneously gained from reports (*a*) giving mean values only, (*b*) not stating the localization of tumours, and (*c*) disregarding the general condition of the patient. Even in the recent report of Seibert *et al.* (1947), who give the mean values from 23 "cancer patients," the average total serum protein was low—6·6 g./100 ml.—but the values ranged up to 8·1 g./100 ml. Gray and Barron (1943) report individual analyses of sera from patients with hepatic tumours. Two out of 5 cases had high total proteins (8·99 and 8·38), two low (6·51 and 5·81), and one case was in the higher range of the normal (7·58). The clinical states of the patients were not mentioned. In an unpublished series of our own observations on the serum proteins of patients undergoing X-ray treatment, normal serum proteins were found in 25 mammary cancer cases, operated or non-operated (mean 7·19, range 6·5–8·0 g./100 ml.). High proteins were noted in two cases of pulmonary tumours (8·6 and 8·4 g./100 ml.) at the beginning of hospitalization, and in both cases the serum protein fell to a low level simultaneously with the clinical deterioration of the patient, reaching values between 5·7 and 5·9 g./100 ml. It seems possible that these findings may explain the discrepancies between the reports on the serum protein concentration of tumour patients by different investigators.

The non-homogeneity of the albumin in normal rat sera at pH 8 has not been previously reported. Electrophoretic analyses of rat sera have been made by Moore (1945) and by Deutsch and Goodloe (1945). These workers used veronal buffer of pH 8·6 and phosphate buffer of pH 7·4 containing 0·15 M NaCl. These conditions do not approximate sufficiently to those of our experiments to allow comparison.

Moore (1945) found none or very little $\alpha$-globulin in the serum from rats of the Long-Evans strain. But this component was present after thyroidectomy (Moore, Levin and Smelser, 1945). Alpha-globulin was present in serum from our normal rats (blackhooded and Wistars), as well as from rats bearing hepatic tumours. The increase of the $\gamma$- and $\beta$-globulins of these rats might be due to an increase in immune bodies frequently associated with these fractions (Tiselius and Kabat, 1939). This explanation would be in accordance with the concept that the increase of the total serum protein in rats developing hepatic tumours is an immunological phenomenon. On the other hand, an increase of the serum lipids, likely to occur when the liver function is impaired, might cause, or contribute to, an increase of refractive area of the globulins. Since other hepatic or systemic diseases of rats were not investigated, we are not in a position to decide whether the changes in the serum proteins are an expression of malignant disease or are due to liver damage generally.

### SUMMARY.

The serum proteins of rats fed pDA increase rapidly at the time of the manifestation of the tumour.

The increase of serum proteins is independent of the size of the tumour. The serum proteins drop to low levels when the health of the animals deteriorates.

In the patterns of the normal rat sera electrolysed in phosphate at pH 8·0 and $\mu$0·1, $\alpha$-, $\beta$- and $\gamma$ globulin regions could be distinguished, and the albumin showed two components.

The rats which developed tumours due to feeding pDA showed a relative and absolute increase in the globulins, mainly in the $\beta$- and $\gamma$ globulins. The concentration of albumin was unchanged, but generally the albumin peak was not resolved into two components.

The possibility of the changes in the serum proteins being an immunological reaction is discussed.

Our thanks are due to Prof. J. R. Marrack for helpful discussion. One of us (C. H.-L.) wishes to thank the British Empire Cancer Campaign for a grant.

### REFERENCES.

DEUTSCH, H. F, AND GOODLOE M. B.—(1945) *J. biol. Chem.*, **161**, 1.

GRAY, S. J., AND BARRON, E. S. G.—(1943) *J. clin. Invest.*, **22**, 191.

HOCH, H., AND MARRACK, J. R.—(1945) *Brit. med. J.*, ii, 876.

LEATHEM, J. H.—(1947) *Proc. Soc. exp. Biol. N.Y.*, **64**, 90.

LIPPMAN, R. W.—(1948) *Ibid.*, **67**, 193.

LONGSWORTH, L. G.—(1946) *Industr. engng. Chem.* (anal. ed), **18**, 219.

METCOFF, J., AND FAVOUR, C. B.—(1944) *Amer. J. Physiol.*, **142**, 94.

MOORE, D. H.—(1945) *J. biol. Chem.*, **161**, 21.

*Idem*, LEVIN, L., AND SMELSER, G. K.—(1945) *Ibid.*, **157**, 723.

PEDERSEN, K. O.—(1940) in Svedberg, T., and Pedersen, K. O.—(1940) 'The Ultracentrifuge.' London (Oxford University Press).

PHILPOT, J. St. L.—(1938) *Nature*, **141**, 283.

SEIBERT, F. B., SEIBERT, M. V., ATNO, A. J., AND CAMPBELL, H. W.—(1947) *J. clin. Invest.*, **26**, 90.

STERN, K., AND WILLHEIM, R.—(1943) 'The Biochemistry of Malignant Tumours.' Brooklyn (Reference Press).

SVENSSON, H.—(1939) *Kolloidzschr.*, **87**, 181.

THOVERT, J.—(1914) *Ann. phys.*, (9) **2**, 369

TISELIUS, A., AND KABAT, E. A.—(1939) *J. exp. Med.*, **69**, 119.